# Neurofibromatosis type 1

**DOI:** 10.1590/0100-3984.2022.55.1e2

**Published:** 2022

**Authors:** Alexandre Dias Mançano

**Affiliations:** Sabin Medicina Diagnóstica, Brasília, DF. Brazil. Email: alex.manzano1@gmail.com.

Neurofibromatosis type 1 (NF1), or von Recklinghausen disease, is one of the most common genetic diseases, with an estimated incidence of 1:2,500 to 1:3,000 people, regardless of gender or ethnicity^(1)^. An extremely interesting piece of data regarding the disease is that in up to 50% of cases the disease manifests as a result of a mutation in the *NF1* gene in individuals with no known family histor^(1)^.

Among patients with NF1, the clinical presentation of the disease varies greatly. The diagnosis is made on the basis of the presence of at least two of the following seven criteria^(2)^: six or more café-au-lait spots; axillary or inguinal ephelides; two or more cutaneous neurofibromas of any type or one plexiform neurofibroma; two or more pigmented iris hamartomas (Lisch nodules); a specific skeletal lesion (sphenoid wing dysplasia or long bone dysplasia); an optic nerve glioma; and at least one first-degree relative affected by the disease.

The *NF1* gene is a tumor suppressor gene that is located on the long arm of chromosome 17. Regular follow-up of patients with NF1 is recommended because of the increased risk of neoplasms, such as optic nerve gliomas, glioblastomas, malignant tumors of peripheral nerves, pheochromocytomas, gastrointestinal stromal tumors, and carcinoid tumors of the duodenum, as well as breast cancer, leukemia, and rhabdomyosarcoma^(1)^. Patients with NF1 can present with thoracic involvement, including intrathoracic neurogenic tumors, meningoceles, kyphoscoliosis, rib deformities, cutaneous or subcutaneous neurofibromas of the chest wall, bullous lung disease, and interstitial lung disease(^3^, ^4^, ^5^).

The article "Neurofibromatosis type 1: evaluation by chest computed tomography", by Alves Júnior et al.^(6)^, published in the previous issue of **Radiologia Brasileira,** revisits the topic in a didactic way, through a retrospective analysis of 14 patients with NF1 and diffuse pulmonary involvement. The authors evaluated six male patients and eight female patients. The sample included only one child. In 11 patients, the presence or absence of respiratory complaints at the time of chest computed tomography was recorded. Of those patients, five had some type of symptom, the most common being cough and dyspnea, and three of those five patients were smokers.

A number of recent studies in the radiology literature of Brazil have highlighted the importance of imaging methods in the assessment of the chest(^7^, ^8^, ^9^, ^10^, ^11^, ^12^). Among the pulmonary findings on computed tomography scans of the chest, Alves Júnior et al.^(6)^ found, in descending order, cysts (in 92.9% of patients), emphysema (in 57.1%), and subpleural bullae (in 42.9%), typically in combination and bilateral. Cutaneous or subcutaneous neurofibromas in the chest wall were also identified in 85.7% of the patients.

Among patients with NF1, smokers and nonsmokers can both present with pulmonary abnormalities(^13^, ^14^, ^15^), cysts and bullae being the most common such abnormalities. It is also noteworthy that pulmonary involvement in NF1 occurs late in the evolution of the disease, typically when patients are in the third or fourth decade of life.

The patient sample evaluated by Alves Júnior et al.^(6)^ included only one child. The reading of this article should be required for thoracic and general radiologists, because the recognition of pulmonary impairment in NF1 can play a fundamental role in the follow-up of these patients. In that study, approximately 45.4% of the patients for whom respiratory symptoms were recorded had some respiratory complaint, although three of them were smokers.

Finally, further studies are needed in order to correlate pulmonary tomography findings with the evolution of respiratory function in patients with NF1. Such studies could also determine the impact that those findings have on survival.

## References

[1] Hirbe AC, Gutmann DH (2014). Neurofibromatosis type 1: a multidisciplinary approach to
care. Lancet Neurol.

[2] No authors listed. Neurofibromatosis (1988). Conference statement. National Institutes of Health Consensus
Development Conference. Arch Neurol.

[3] Ryu JH, Parambil JG, McGrann PS (2005). Lack of evidence for an association between neurofibromatosis and
pulmonary fibrosis. Chest.

[4] Irion KL, Gasparetto TD, Marchiori E (2008). Neurofibromatosis type 1 with tracheobronchial neurofibromas:
case report with emphasis on tomographic findings. J Thorac Imaging.

[5] Prudhomme L, Delleci C, Trimouille A (2020). Severe thoracic and spinal bone abnormalities in
neurofibromatosis type 1. Eur J Med Genet.

[6] Alves SF, Irion KL, Melo ASA (2021). Neurofibromatose tipo 1: estudo por tomografia computadorizada do
tórax. Radiol Bras.

[7] Barbosa PNVP, Bitencourt AGV, Miranda GD (2020). Chest CT accuracy in the diagnosis of SARS-CoV-2 infection:
initial experience in a cancer center. Radiol Bras.

[8] Louza GF, Nobre LF, Mançano AD (2020). Lymphocytic interstitial pneumonia: computed tomography findings
in 36 patients. Radiol Bras.

[9] Farias LPG, Strabelli DG, Fonseca EKUN (2020). Thoracic tomographic manifestations in symptomatic respiratory
patients with COVID-19. Radiol Bras.

[10] Mogami R, Lopes AJ, Araújo RC (2021). Chest computed tomography in COVID-19 pneumonia: a retrospective
study of 155 patients ata university hospital in Rio de Janeiro,
Brazil. Radiol Bras.

[11] Ando SM, Fonseca EKUN, Frassei JS (2021). The role of the radiologist in the assessment of thoracic changes
after radiotherapy. Radiol Bras.

[12] Di Puglia EBM, Rodrigues RS, Daltro PA (2021). Tomographic findings in bronchial atresia. Radiol Bras.

[13] Ueda K, Honda O, Satoh Y (2015). Computed tomography (CT) findings in 88 neurofibromatosis 1 (NF1)
patients: prevalence rates and correlations of thoracic
findings. Eur J Radiol.

[14] Zamora AC, Collard HR, Wolters PJ (2007). Neurofibromatosis-associated lung disease: a case series and
literature review. Eur Respir J.

[15] Oikonomou A, Vadikolias K, Birbilis T (2011). HRCT findings in the lungs of non-smokers with
neurofibromatosis. Eur J Radiol.

